# Optimal coordination of maximal-effort horizontal and vertical jump motions – a computer simulation study

**DOI:** 10.1186/1475-925X-6-20

**Published:** 2007-06-01

**Authors:** Akinori Nagano, Taku Komura, Senshi Fukashiro

**Affiliations:** 1 Institute of Medical Sciences, University of Aberdeen, Aberdeen, UK; 2 Computational Biomechanics Unit, RIKEN, Saitama, Japan; 3 School of Informatics, University of Edinburgh, Edinburgh, UK; 4 Graduate School of Interdisciplinary Information Studies, University of Tokyo, Tokyo, Japan; 5 Department of Life Sciences (Sports Sciences), University of Tokyo, Tokyo, Japan

## Abstract

**Background:**

The purpose of this study was to investigate the coordination strategy of maximal-effort horizontal jumping in comparison with vertical jumping, using the methodology of computer simulation.

**Methods:**

A skeletal model that has nine rigid body segments and twenty degrees of freedom was developed. Thirty-two Hill-type lower limb muscles were attached to the model. The excitation-contraction dynamics of the contractile element, the tissues around the joints to limit the joint range of motion, as well as the foot-ground interaction were implemented. Simulations were initiated from an identical standing posture for both motions. Optimal pattern of the activation input signal was searched through numerical optimization. For the horizontal jumping, the goal was to maximize the horizontal distance traveled by the body's center of mass. For the vertical jumping, the goal was to maximize the height reached by the body's center of mass.

**Results:**

As a result, it was found that the hip joint was utilized more vigorously in the horizontal jumping than in the vertical jumping. The muscles that have a function of joint flexion such as the m. iliopsoas, m. rectus femoris and m. tibialis anterior were activated to a greater level during the countermovement in the horizontal jumping with an effect of moving the body's center of mass in the forward direction. Muscular work was transferred to the mechanical energy of the body's center of mass more effectively in the horizontal jump, which resulted in a greater energy gain of the body's center of mass throughout the motion.

**Conclusion:**

These differences in the optimal coordination strategy seem to be caused from the requirement that the body's center of mass needs to be located above the feet in a vertical jumping, whereas this requirement is not so strict in a horizontal jumping.

## Background

To date, jumping motions have been studied by many researchers in the field of biomechanics. One of the major purposes of those preceding studies was to investigate the coordination strategy of the human body during explosive activities. Interesting findings have been reported in numerous studies [1-5], which utilized various forms of vertical jump motions as the subject. There are several major reasons why vertical jump motions have been studied so frequently. One of them is that vertical jump motions are frequently performed in sports activities. Good examples can be found in such sports as volleyball, basketball and so on [6,7]. Therefore it is practically valuable to investigate the mechanism of vertical jump motions.

However, from the viewpoint of sports biomechanics, it is also valuable to investigate the motion of the body in the horizontal direction during jumping. This is because it is often important to maximize the horizontal distance of jumping in sports activities. Long jump in track and field is an obvious example [8,9]. Even in other sports such as volleyball and basketball, athletes typically do not simply jump up vertically but generate a certain amount of horizontal momentum in order to achieve a good overall performance (spiking, shooting etc.). Therefore it is valuable to examine the mechanism of jumping motions with a consideration of the horizontal component (horizontal jumping).

Herzog (1986) evaluated the contribution of various body segments to the maintenance of body orientation during the flight phase of horizontal jumping [10]. Robertson and Fleming (1987) compared the kinetics of standing horizontal and vertical jumping motions [11]. Fukashiro et al. (2005) compared the kinematics, kinetics (joint moment and power) and electromyography of maximal-effort horizontal and vertical jump motions [12]. It was found that the trunk segment was placed in the forward direction at the time of take off in the horizontal jump. It was also reported that there was a marked difference in the activation pattern of biarticular muscles. Many preceding studies including these ones have used the methodology of experimental data collection and analysis through which interesting findings have been obtained.

However, especially when utilizing human subjects, there exists a major limitation associated with the experimental methodology: it is extremely difficult to perform direct measurements of such essential variables as muscle forces and its length change under experimental settings [13-15]. As this limitation seems almost unavoidable, it is worthwhile to use an approach to circumvent this problem. The methodology of computer simulation has a potential to provide a solution for this problem [16,17]. Using this methodology, it is possible to investigate the detailed behavior of individual muscles and other components of the musculoskeletal system assuming that the simulation model sufficiently captures the fundamental nature of the human body.

Ridderikhoff et al. (1999) generated a horizontal jumping motion using computer simulation [18]. A whole body musculoskeletal model containing six lower limb muscles (mm. glutei, hamstrings, m. rectus femoris, mm. vasti, m. gastrocnemius, m. soleus) was utilized. The motions were compared between squat vertical and horizontal jumping. For the horizontal jumping, an initial angular velocity (0.6 rad/s) was assigned to the body segments at the start of a simulation. Thereafter, the profile of activation timing of the six lower limb muscles was modified through numerical optimization with a goal of maximizing the horizontal distance traveled by the body's center of mass. Although this study [18] was an innovative one that investigated the mechanism of horizontal jumping using computer simulation, the model was not allowed to make a countermovement during the jumping. Considering the fact that a countermovement enhances jumping performance [19], it would be valuable to investigate vertical and horizontal jumping motions with a countermovement.

To increase the knowledge regarding the mechanism of vertical and horizontal jumping motions, we aimed at simulating these jumping motions with a countermovement, from an identical initial posture (angles as well as angular velocities of all segments). This can be accomplished by initiating a simulation from an identical upright standing posture and finding the optimal profiles of activation input signal that generate countermovement jumping motions. The purpose of this study was to compare the optimal coordination of a countermovement horizontal jump and a countermovement vertical jump starting from an identical initial posture.

## Methods

**Figure 1:**
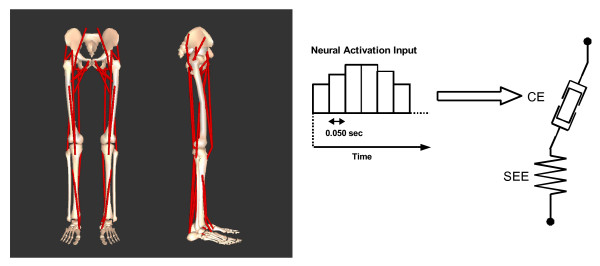
**The musculoskeletal model developed for this study**. The bones in the leg and leg muscles are shown. The HAT segment includes the head, arms and trunk segments in a single rigid body.

A 3D simulation model of the human body was developed using DADS-3D (LMS CADSI, Coralville, Iowa, USA) with the FORTRAN-based USER.FORCE option. The skeletal model consisted of nine rigid body segments (head-arms-trunk (HAT) segment, right and left upper leg segments, right and left lower leg segments, right and left feet segments and right and left toe segments) connected with frictionless joints (Figure 1) [20,21]. Body segmental parameter values were derived from an anthropometric study that utilized human subjects [22] (body mass = 73.1 kg). Hip joints were modeled as ball and socket joints that have three degrees of freedom (flexion/extension, abduction/adduction, internal/external rotation). Knee joints were modeled as hinge joints (flexion/extension). Ankle joints were modeled as biaxial joints with tilted axes as reported in [23] (dorsi/plantar flexion, inversion/eversion). Metatarsophalangeal joints were modeled as hinge joints (flexion/extension). Therefore the number of degrees of freedom of the joints was 14. By adding the degrees of freedom of the whole body position (3) and orientation (3), the total number of degrees of freedom was 20.

Thirty-two Hill-type [24] lower limb muscles (sixteen muscles in each leg) were implemented into the skeletal model (Table 1) [20,21]. These include most of major muscles found in the human leg. Muscle parameter values, i.e., optimal contractile element length (L_CEopt_), maximal isometric force of the contractile element (F_max_), pennation angle (α_pen_) and unloaded length of the series elastic element (L_slack_), were derived from [25] and [26]. The specific tension value of 31.5 N/cm^2 ^[27] was adopted. Muscles that have similar biomechanical function were merged to compose a muscle group. For example, m. vastus medialis, m. vastus intermedialis and m. vastus lateralis were merged as a single mm. vasti. This step was necessary in order to maintain the complexity of the model within a manageable level. Muscles or muscle groups whose maximal isometric force is greater than 500 N were implemented in the musculoskeletal model. M. biceps femoris short head, whose F_max _is smaller than 500 N, was also implemented as the only mono-articular knee flexor muscle (Table 1). The coordinates of the origin, insertion and via-points of these muscles were derived from [25]. A bilateral symmetry was assumed between the sides of the body (i.e., the right side is a mirror image of the left side). A Hill-type muscle-tendon complex was composed of a contractile element (CE) and a series elastic element (SEE) serially connected with a pennation angle (Figure 1). The mathematical model of the contractile element represented the force-length-velocity relations. Passive stress-strain property of the series elastic element was modeled with a quadratic function that represents experimentally collected stress-strain property of tendons. The strain of the SEE was 4% when the CE was developing a maximal isometric force. A detailed mathematical representation of these components can be found in [28].

**Table 1:** The muscle parameter values used in this study

	F_max _(N)	L_CEopt _(m)	α_pen _(deg)	L_slack _(m)
ILIOP	1544	0.104	8	0.130
GMAXI	1883	0.142	5	0.125
GMEDI	1966	0.054	8	0.078
GMINI	849	0.038	1	0.051
ADDLO	716	0.138	6	0.110
ADDMA	1916	0.087	5	0.060
ADDBR	531	0.133	0	0.020
HEXRO	1512	0.054	0	0.024
RECTF	1353	0.084	5	0.432
HAMST	3054	0.080	15	0.359
VASTI	6718	0.087	3	0.315
BFESH	256	0.173	23	0.100
GASTR	2044	0.045	17	0.408
TIBAN	532	0.098	5	0.223
SOLEU	5881	0.030	25	0.268
OPFLE	3137	0.031	12	0.310

The values for each muscle are shown. F_max_: Maximal isometric force of the contractile element. L_CEopt_: Optimal length of the contractile element. α_pen_: Pennation angle. L_slack_: Slack (unloaded) length of the series elastic element. ILIOP: m. iliopsoas. GMAXI: m. gluteus maximus. GMEDI: m. gluteus medius. GMIN: m. gluteus minimus. ADDLO: m. adductor longus. ADDMA: m. adductor magnus. ADDBR: m. adductor brevis. HEXRO: merged hip external rotator muscles. RECTF: m. rectus femoris. HAMST: merged hamstrings. VASTI: mm. vasti. BFESH: m. biceps femoris short head. GASTR: m. gastrocnemius. TIBAN: m. tibialis anterior. SOLEU: m. soleus. OPFLE: merged monoarticular planter flexor muscles other than m. soleus.

The neural activation input signal to individual muscles was represented by a series of step functions with duration of 0.050 s [29] (Figure 1). The excitation dynamics of the contractile element was modeled with a first-order ordinary differential equation as described in [30]. The muscles started their action from an initial activation level, and changed the activation level as directed by the input signal. The non-linear repulsive interaction between a foot segment and the ground was modeled with five points similar to [31]. The non-linear passive joint properties that function to limit the joint range of motion were adopted from [31].

Maximal-effort horizontal and vertical countermovement jumping motions were generated through forward dynamic computer simulation and numerical optimization. A simulation was initiated from a static (no motions) upright posture with the hip, knee and ankle joints slightly flexed (5 degrees: dorsiflexed for the ankle joint) to facilitate the generation of a countermovement. The maximal simulation time was set as 1.2 s, which is more than 20% greater than experimentally observed movement times for these motions [12]. The simulation model and the numerical optimization algorithm were free to choose the optimal take-off time for each jumping between 0.0 s and 1.2 s. Muscle activation input profiles including the initial level of muscle activation were modified through numerical optimization [32]. The projectile motion of the body's center of mass after the instant of take-off was evaluated. The trajectory of the body's center of mass can be represented as:

*X *= *X*_*t.o*. _+ *V*_*Xt.o*._·*t*

Y=Yt.o.+VYt.o.⋅t−12⋅g⋅t2
 MathType@MTEF@5@5@+=feaafiart1ev1aaatCvAUfKttLearuWrP9MDH5MBPbIqV92AaeXatLxBI9gBaebbnrfifHhDYfgasaacH8akY=wiFfYdH8Gipec8Eeeu0xXdbba9frFj0=OqFfea0dXdd9vqai=hGuQ8kuc9pgc9s8qqaq=dirpe0xb9q8qiLsFr0=vr0=vr0dc8meaabaqaciaacaGaaeqabaqabeGadaaakeaacqWGzbqwcqGH9aqpcqWGzbqwdaWgaaWcbaGaemiDaqNaeiOla4Iaem4Ba8MaeiOla4cabeaakiabgUcaRiabdAfawnaaBaaaleaacqWGzbqwcqWG0baDcqGGUaGlcqWGVbWBcqGGUaGlaeqaaOGaeyyXICTaemiDaqNaeyOeI0YaaSaaaeaacqaIXaqmaeaacqaIYaGmaaGaeyyXICTaem4zaCMaeyyXICTaemiDaq3aaWbaaSqabeaacqaIYaGmaaaaaa@4C3B@

where t, g, X_t.o._, Y_t.o._, V_Xt.o. _and V_Yt.o. _represent time after the instant of take-off, acceleration due to gravity (9.8 m/s^2^), X (horizontal) and Y (vertical) position of the body's center of mass at the instant of take-off and X and Y velocity of the body's center of mass at the instant of take-off, respectively. Assuming that the posture of the whole body at the instant of take-off and the posture at the instant of landing are identical, the flight time of this projectile motion (t_flight_) is calculated as:

tflight=2⋅VYt.o.g
 MathType@MTEF@5@5@+=feaafiart1ev1aaatCvAUfKttLearuWrP9MDH5MBPbIqV92AaeXatLxBI9gBaebbnrfifHhDYfgasaacH8akY=wiFfYdH8Gipec8Eeeu0xXdbba9frFj0=OqFfea0dXdd9vqai=hGuQ8kuc9pgc9s8qqaq=dirpe0xb9q8qiLsFr0=vr0=vr0dc8meaabaqaciaacaGaaeqabaqabeGadaaakeaacqWG0baDdaWgaaWcbaGaemOzayMaemiBaWMaemyAaKMaem4zaCMaemiAaGMaemiDaqhabeaakiabg2da9maalaaabaGaeGOmaiJaeyyXICTaemOvay1aaSbaaSqaaiabdMfazjabdsha0jabc6caUiabd+gaVjabc6caUaqabaaakeaacqWGNbWzaaaaaa@4374@

Therefore, the horizontal position of the body's center of mass at the instant of landing (X_landing_) is calculated as:

Xlanding=Xt.o.+VXt.o.⋅2⋅VYt.o.g
 MathType@MTEF@5@5@+=feaafiart1ev1aaatCvAUfKttLearuWrP9MDH5MBPbIqV92AaeXatLxBI9gBaebbnrfifHhDYfgasaacH8akY=wiFfYdH8Gipec8Eeeu0xXdbba9frFj0=OqFfea0dXdd9vqai=hGuQ8kuc9pgc9s8qqaq=dirpe0xb9q8qiLsFr0=vr0=vr0dc8meaabaqaciaacaGaaeqabaqabeGadaaakeaacqWGybawdaWgaaWcbaGaemiBaWMaemyyaeMaemOBa4MaemizaqMaemyAaKMaemOBa4Maem4zaCgabeaakiabg2da9iabdIfaynaaBaaaleaacqWG0baDcqGGUaGlcqWGVbWBcqGGUaGlaeqaaOGaey4kaSIaemOvay1aaSbaaSqaaiabdIfayjabdsha0jabc6caUiabd+gaVjabc6caUaqabaGccqGHflY1daWcaaqaaiabikdaYiabgwSixlabdAfawnaaBaaaleaacqWGzbqwcqWG0baDcqGGUaGlcqWGVbWBcqGGUaGlaeqaaaGcbaGaem4zaCgaaaaa@5502@

This variable X_landing _was taken as the performance criterion (objective function) for a horizontal jumping motion. This approach allows an evaluation of the horizontal distance traveled by the body's center of mass by the time its vertical position comes back to the same height as the instant of take-off. In a vertical jumping, the peak height reached by the body's center of mass after jumping up can be calculated as:

Ypeak=Yt.o.+VYt.o.      22⋅g
 MathType@MTEF@5@5@+=feaafiart1ev1aaatCvAUfKttLearuWrP9MDH5MBPbIqV92AaeXatLxBI9gBamXvP5wqSXMqHnxAJn0BKvguHDwzZbqegyvzYrwyUfgarqqtubsr4rNCHbGeaGqiA8vkIkVAFgIELiFeLkFeLk=iY=Hhbbf9v8qqaqFr0xc9pk0xbba9q8WqFfeaY=biLkVcLq=JHqVepeea0=as0db9vqpepesP0xe9Fve9Fve9GapdbaqaaeGacaGaaiaabeqaamqadiabaaGcbaGaemywaK1aaSbaaSqaaiabdchaWjabdwgaLjabdggaHjabdUgaRbqabaGccqGH9aqpcqWGzbqwdaWgaaWcbaGaemiDaqNaeiOla4Iaem4Ba8MaeiOla4cabeaakiabgUcaRmaalaaabaGaemOvay1aa0baaSqaaiabbMfazjabbsha0jabb6caUiabb+gaVjabb6caUaqaaiabbccaGiabbccaGiabbccaGiabbccaGiabbccaGiabbccaGiabbkdaYaaaaOqaaiabikdaYiabgwSixlabdEgaNbaaaaa@5D32@

This variable Y_peak _was taken as the performance criterion for a vertical jumping motion. These criterion variables X_landing _and Y_peak _were maximized through the numerical optimization to simulate maximal-effort jumping. This is equivalent to instructing human subjects to jump "forward as far as possible" and "upward as high as possible", respectively, as has been conducted in [12]. When human subjects are asked to jump as forward as possible, they would not only try to maximize the momentums, but also try to configure their legs to place their feet as far as possible. Considering the length of the human leg, the technique of placing the feet as far as possible can not be ignored in experimental settings. However, this was not taken into consideration in this study, in order to focus the analysis and discussion on the kinetics until the instant of take off. The objective function used in this study would be a reasonable measure for considering the horizontal and vertical momentums and the translational mechanical energy given to the body's center of mass by the time of take off. Similarly, the angular momentum of segments was not considered as a part of the objective function. This is because the magnitude of the angular component of mechanical energy (0.5*(moment of inertia)*(angular velocity)^2) had been found to be rather small through pilot calculations. Optimal activation input profiles for individual muscles were searched. The optimization process was terminated when the objective function value had not improved for 10,000 successive iterations, which corresponds to approximately 60,000 function evaluations without any improvement.

## Results

**Figure 2:**
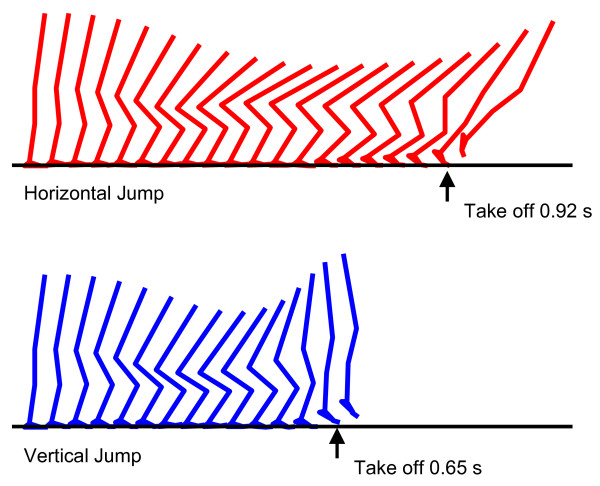
**The kinematics of the horizontal jumping and the vertical jumping generated in this study (sagittal view)**. The horizontal jumping kinematics is shown in the top, and the vertical jumping kinematics is shown in the bottom.

A smooth horizontal jumping motion and a smooth vertical jumping motion with a countermovement were generated as results of the numerical optimization (Figure 2). The duration from the start of a motion (simulation) through the instant of take-off was 0.92 s and 0.65 s for the horizontal jump and for the vertical jump, respectively. These motions were completed within the maximal simulation time adopted in this study (1.2 s). The maximal horizontal distance reached by the body's center of mass in the horizontal jump was 1.238 m measured from the initial starting posture (Table 2). The maximal height reached by the body's center of mass in the vertical jump was 1.316 m measured from the floor. This corresponds to a jumping height of 0.385 m, as the initial height of the body's center of mass was 0.931 m above the floor (Table 2). The total amount of translational mechanical energy (mass*gravity*height+0.5*(mass)*(velocity)^2) gain of the body's center of mass throughout the horizontal jumping motion was 258.9 J, whereas this parameter for the vertical jumping motion was 180.3 J. The angular component of mechanical energy was found to be small compared to these total amounts (less than 6% and 3% for horizontal and vertical jumping motions, respectively).

**Table 2:** The characteristics of the optimal jumping motions obtained through numerical optimization

	X_ini _(m)	Y_ini _(m)	X_t.o. _(m)	Y_t.o. _(m)	V_Xt.o. _(m/s)	V_Yt.o. _(m/s)	V_XYt.o. _(m/s)	X_max _(m)	Y_max _(m)	E_gain _(J)
HJ	0.0	0.931	0.568	0.899	2.201	1.493	2.660	1.238	1.013	258.9
VJ	0.0	0.931	0.010	1.066	-0.063	2.218	2.219	-0.019	1.316	180.3

X_ini_: Initial horizontal position of the body's center of mass. Y_ini_: Initial vertical position of the body's center of mass measured from the floor. X_t.o._: Horizontal position of the body's center of mass at the instant of take-off. Y_t.o._: Vertical position of the body's center of mass at the instant of take-off measured from the floor. V_Xt.o._: Horizontal velocity of the body's center of mass at the instant of take-off. V_Yt.o._: Vertical velocity of the body's center of mass at the instant of take-off. V_XYt.o._: Resultant velocity of the body's center of mass at the instant of take-off. X_max_: Maximal distance traveled by the body's center of mass. (Note: this value was negative in the vertical jumping, as the model jumped up slightly backwards.) Y_max_: Maximal height reached by the body's center of mass measured from the floor. E_gain_: Energy gain of the body's center of mass throughout the jumping motion.

**Figure 3:**
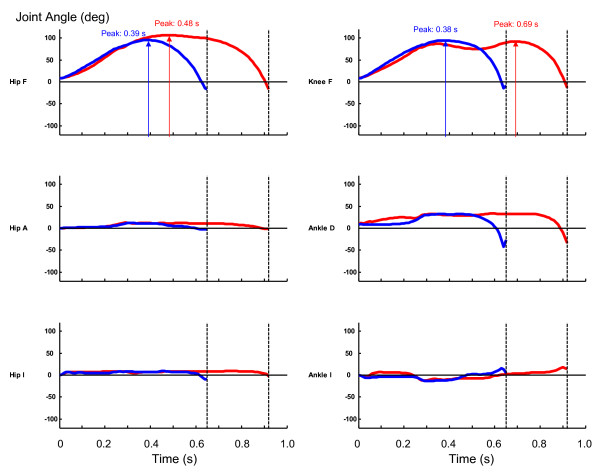
**The profiles of the joint angles**. The red curves represent the profiles for the horizontal jumping. The blue curves represent the profiles for the vertical jumping. Hip F: hip joint flexion (peaked at 0.48 s and 0.39 s for horizontal jump and vertical jump, respectively); Hip A: hip joint adduction; Hip I: hip joint internal rotation; Knee F: knee joint flexion (peaked at 0.69 s and 0.38 s for horizontal jump and vertical jump, respectively); Ankle D: ankle joint dorsiflexion; Ankle I: ankle joint inversion.

The magnitude of hip joint flexion was greater in the horizontal jump than in the vertical jump throughout the motion (Figure 3). The magnitude of hip joint adduction/abduction and hip joint internal/external rotation was small both in the horizontal jump and in the vertical jump. The magnitude of knee joint flexion was similar between the horizontal jump and the vertical jump. The peak value of ankle joint dorsiflexion was similar between the horizontal jump and the vertical jump, although the ankle joint assumed a dorsiflexed posture earlier and was kept dorsiflexed for a longer duration in the horizontal jump than in the vertical jump. The amount of ankle inversion was small in both types of jumping motions.

**Figure 4:**
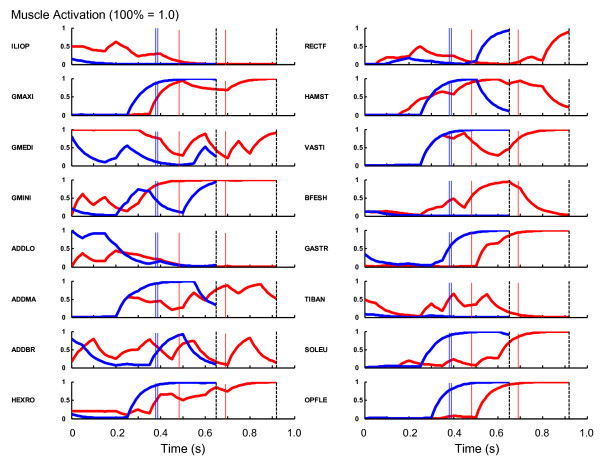
**The profiles of the muscle activation**. The red curves represent the profiles for the horizontal jumping. The blue curves represent the profiles for the vertical jumping. The dashed vertical lines represent the instant of take-off. The thin red and blue lines correspond to the instances when the hip and knee joints attained the peak flexion values (Figure 3).

The profiles of the optimal muscle activation were also markedly different between the horizontal jump and the vertical jump (Figure 4). A large activation of the m. iliopsoas was observed in the horizontal jump, whereas there was a relatively small activation of this muscle during the vertical jump. This finding was evident from the moment of initiation of the movement. The gluteal muscles (m. gluteus maximus, m. gluteus medius and m. gluteus minimus) were activated for a longer duration in the horizontal jump than in the vertical jump. The m. rectus femoris was activated to a greater level during the countermovement phase in the horizontal jump than in the vertical jump. The activation level of the mm. vasti experienced a drop during the push-off phase in the horizontal jump, although the level was almost consistently ~ 100% during the push-off phase in the vertical jump. A large activation of the m. biceps femoris short head and m. tibialis anterior was observed during the push-off phase in the horizontal jump.

**Figure 5:**
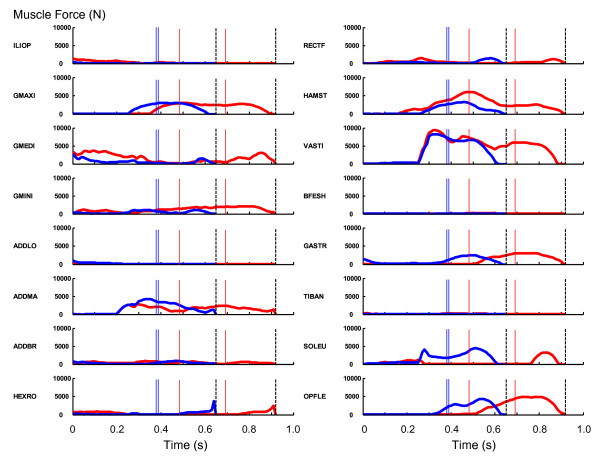
**The profiles of muscle force output**. The red curves represent the profiles for the horizontal jumping. The blue curves represent the profiles for the vertical jumping. The dashed vertical lines represent the instant of take-off. The added values for two contralateral muscles are shown. The thin red and blue lines correspond to the instances when the hip and knee joints attained the peak flexion values (Figure 3).

The differences in muscle activation profiles between the horizontal jump and the vertical jump were also reflected in the muscle force development profiles (Figure 5). The muscle force development of the m. iliopsoas was greater in the horizontal jump than in the vertical jump. Once again, this was evident from the moment of initiation of the movement. The force development of the gluteal muscles was more pronounced in the horizontal jump than in the vertical jump. The force development of the hamstrings was also more pronounced in the horizontal jump than in the vertical jump.

The amount of mechanical work output of muscles was generally similar between the horizontal jump and the vertical jump with a few exceptions (Table 3). Specifically, the m. iliopsoas, hip external rotator muscles and hamstrings were the only exceptions in which the difference was greater than 10 J. Generally speaking, the work output of the muscles was smaller in the horizontal jumping than in the vertical jumping. The total amount of muscle work outputs for all muscles was 357.3 J for the horizontal jumping and 375.8 J for the vertical jumping.

**Table 3:** The work outputs of individual muscles

	Muscle Work (J)		
	HJ	VJ	Δ
ILIOP	36.9	2.0	34.9
GMAXI	49.1	47.9	1.2
GMEDI	7.9	7.7	0.3
GMINI	-4.0	4.5	-8.5
ADDLO	2.1	11.5	-9.4
ADDMA	9.8	9.1	0.7
ADDBR	-4.0	2.5	-6.5
HEXRO	5.3	19.3	-14.1
RECTF	14.1	14.4	-0.3
HAMST	-4.8	10.2	-15.1
VASTI	132.3	134.8	-2.5
BFESH	-1.5	0.3	-1.8
GASTR	29.1	32.0	-2.9
TIBAN	2.6	-0.5	3.1
SOLEU	30.6	28.6	2.0
OPFLE	51.9	51.4	0.5

The added values for two legs (two contralateral muscles) are shown. HJ: Horizontal jumping. VJ: Vertical jumping. Δ: The difference between the values for the horizontal jumping and the vertical jumping.

## Discussion

The purpose of this study was to investigate the coordination strategy of maximal-effort horizontal jumping motion in comparison with vertical jumping motion. A horizontal jumping and a vertical jumping with a countermovement were generated using the technique of computer simulation and numerical optimization (Figure 2, Table 2). It was found that the motion of the hip joint was greater in the horizontal jump than in the vertical jump (Figure 2, 3). This observation is consistent with the findings reported in [12] as well as in [18]. In vertical jumping motions, the orientation of the trunk segment has to be near straight and its angular momentum has to be reduced to near zero at the instant of take-off. This condition is required for the human body to jump up vertically with a straight posture [33,34]. Therefore a smaller action of the hip joint is allowed in a vertical jump than in a horizontal jump.

The optimal movement time for the horizontal jump was greater than that for the vertical jump (0.92 s and 0.65 s, respectively). This result is consistent with the finding reported in [12], in which longer movement duration of the trunk segment was observed in horizontal jump than in vertical jump. These movement times were similar to the ones observed in the experimental study [12], which also suggest that the simulation model and the optimization method employed in this study capture the fundamental nature of human jumping motions.

The magnitude of hip joint flexion during the countermovement was greater in the horizontal jumping than in the vertical jumping (Figure 3). The ankle joint assumed a dorsiflexed posture earlier in the horizontal jumping than in the vertical jumping. Combining these two conditions, the whole body was tilted more in the forward direction in the horizontal jump than in the vertical jump (Figure 2). This result is completely consistent with the experimental observation reported in [12]. This is reasonable considering that it is required to generate a momentum in both forward and upward directions by the instant of take-off in a horizontal jumping. On the other hand, it is required to generate only an upward momentum in a vertical jump. In order to jump upwards with a straight posture, the position of the body's center of mass has to be kept over the feet in a vertical jump. The motions of the hip, knee and ankle joints were coordinated in the vertical jumping to meet this requirement (Figure 2).

When examining the muscle activation (Figure 4) and force development (Figure 5) profiles, it can be observed that the flexor muscles of the leg were recruited to generate greater joint flexion motions during the countermovement phase in the horizontal jumping. This phenomenon was pronounced in the action of the m. iliopsoas, m. rectus femoris and m. tibialis anterior. This was evident from the moment of initiation of the motion. This action had an effect of moving the body's center of mass in the forward direction during the countermovement. This configuration of body segments helped enhance the horizontal momentum delivered to the body's center of mass through the countermovement. The duration of activation of the hip joint extensor muscles (the gluteal muscles and hamstrings) was longer in the horizontal jump than in the vertical jump. This observation is consistent with the finding that the hip joint was utilized more vigorously in the horizontal jump than in the vertical jump. There was a drop in the activation of the mm. vasti during the push-off phase, whereas there was a great activation of the m. biceps femoris during the push-off phase in the horizontal jump. This coordination was needed in order to maintain the forward inclined posture during the push-off phase.

The work outputs of the individual muscles (Table 3) were generally similar between the horizontal jump and the vertical jump. The m. iliopsoas, hip external rotator muscles and hamstrings were the only exceptions in which more than 10 J of difference was observed in the work output. The work output of the m. iliopsoas was greater in the horizontal jumping than in the vertical jumping because this muscle was activated to a greater level during the countermovement in the horizontal jumping (Figures 4 and 5). On the other hand, the work output of the hip external rotator muscles and hamstrings was smaller in the horizontal jumping than in the vertical jumping. Especially, the work output of the hamstrings was negative in the horizontal jumping (-4.8 J, Table 3). This suggests that the hamstrings experienced an eccentric action in which this muscle was stretched at the same time as exerting a muscle force. This is because a great momentum was given to the trunk segment throughout the countermovement in the direction of hip joint flexion, and the hamstrings was utilized to counteract the momentum. Therefore, the force output of this muscle was great (Figure 5) and the work output was negative (Table 3). A similar mechanism seems to have caused the smaller work output of the hip external rotator muscles in the horizontal jump (5.3 J) than in the vertical jump (19.3 J).

It is interesting that the amount of translational mechanical energy gain of the body's center of mass throughout the jumping motion was greater in the horizontal jump (258.9 J) than in the vertical jump (180.3 J; 70% as compared to in the horizontal jump). This result is consistent with what has been reported in [12], in which the translational energy gain of the body's center of mass during a vertical jump was as much as 64% of the translational energy gain of the body's center of mass during a horizontal jump. As there was only a minor difference in the total muscle work output (the total value was 357.3 J for the horizontal jumping and 375.8 J for the vertical jumping; the difference was only 5%), it was suggested that muscular work was transferred to the mechanical energy of the body's center of mass more effectively through the horizontal jumping than through the vertical jumping. An explanation for this finding is in the difference of transfer of mechanical energy during the countermovement. In the horizontal jumping, a reduction of potential energy as the body segments were moved to a lower position was coupled with an increase of kinetic energy of those segments moving in the forward direction (Figure 2). Therefore, there was a smaller loss of mechanical energy during the countermovement in the horizontal jumping. However, in the vertical jumping, all the downward momentum generated during the countermovement had to be cancelled by muscular efforts before the body started moving upward (Figure 2). Therefore, there was a greater energy loss during the countermovement. The contribution of the angular component of the mechanical energy (0.5*(moment of inertia)*(angular velocity)^2) was rather small in both types of jumping at the instant of take off. The magnitude was less than 6% for horizontal jumping, and less than 3% for vertical jumping. In other words, the translational components had much greater contributions. This might be because the value of moment of inertia of human body segments is generally small.

In this study, the resultant velocity of the body's center of mass at the instant of take-off was greater in the horizontal jumping than in the vertical jumping (Table 2). This result seems to be inconsistent with the results reported in [18], in which the resultant velocity of the body's center of mass at the instant of take-off was almost identical between a horizontal jump and a vertical jump. This difference can be explained by the existence/absence of a countermovement. As the jumping motion simulated in this study employed a countermovement, and as an effective transfer of mechanical energy was observed in the horizontal jumping, the body's center of mass experienced a greater gain of mechanical energy by the instant of take-off. It is suggested that this mechanism of energy transfer was less evident in the horizontal jumping motion studied in [18], as that motion was a squat jumping instead of a countermovement jumping.

The optimal angle of projection obtained in this study was 34 deg, whereas this parameter obtained in a preceding experimental study was 48 deg [12]. This discrepancy can be explained by the difference in the musculoskeletal properties of the model and the subjects, i.e., the subjects that participated in [12] were 'stronger' than the model utilized in this study. This is evident by comparing the vertical jumping height obtained in this study (38.5 cm) with the vertical jumping height of the subjects (41.0 cm), although both of these figures are within the range of experimental observations reported in numerous preceding studies on vertical jumping. This difference seems reasonable considering that the subjects were trained athletes (Australian Football) in [12]. As discussed previously, the horizontal distance traveled in the horizontal jump was calculated as

Xlanding=Xt.o.+VXt.o.⋅2⋅VYt.o.g
 MathType@MTEF@5@5@+=feaafiart1ev1aaatCvAUfKttLearuWrP9MDH5MBPbIqV92AaeXatLxBI9gBaebbnrfifHhDYfgasaacH8akY=wiFfYdH8Gipec8Eeeu0xXdbba9frFj0=OqFfea0dXdd9vqai=hGuQ8kuc9pgc9s8qqaq=dirpe0xb9q8qiLsFr0=vr0=vr0dc8meaabaqaciaacaGaaeqabaqabeGadaaakeaacqWGybawdaWgaaWcbaGaemiBaWMaemyyaeMaemOBa4MaemizaqMaemyAaKMaemOBa4Maem4zaCgabeaakiabg2da9iabdIfaynaaBaaaleaacqWG0baDcqGGUaGlcqWGVbWBcqGGUaGlaeqaaOGaey4kaSIaemOvay1aaSbaaSqaaiabdIfayjabdsha0jabc6caUiabd+gaVjabc6caUaqabaGccqGHflY1daWcaaqaaiabikdaYiabgwSixlabdAfawnaaBaaaleaacqWGzbqwcqWG0baDcqGGUaGlcqWGVbWBcqGGUaGlaeqaaaGcbaGaem4zaCgaaaaa@5502@

By analyzing the right-hand side of this formula, it can be derived that the second term is maximized when the projection angle is 45 deg. However, the first term (X_t.o._) also made a substantial contribution in this study. The computer simulation model and the numerical optimization chose the strategy of increasing X_t.o. _with a smaller angle of projection. Although it is assumed that the optimal projection angle will become closer to 45 deg when the muscular parameters of the model are strengthened, customizing a computer simulation model to a specific subject population requires very complex and sophisticated treatments. This issue needs to be addressed in future studies.

In this study, the optimal pattern of muscle activation, including the initial muscle activation level, was searched using numerical optimization. This resulted in reasonable movements both for horizontal and vertical jumping motions. It is observed that the initial level of muscle activation and muscle forces were not identical between these motions (Figure 4 and 5). This result, i.e., the discrepancies in the initial conditions of the simulation, might seem controversial at a first glance. However, these initial conditions were not *given *to the model: instead, the numerical optimization procedure found these initial conditions to be the most suitable for the model to perform jumping starting from the identical upright posture. We believe the general similarity between the simulated body dynamics and that of the human subjects suggest the validity of the approach taken in this study. As the objective function utilized in this study considered only the translational motions of the body's center of mass, rotational component of the mechanical energy was not explicitly analyzed. Techniques of foot placement at the time of landing were not discussed either. However, these might become more important when performing more precise comparisons between the simulated and experimentally captured motions, or when applying the methods and findings of this study to sports scenes. More sophisticated modeling and simulation that include the landing phase will be valuable in future studies with a goal of reducing the risk of injuries in athletes.

## Conclusion

To conclude, the differences of the coordination strategy of maximal-effort horizontal and vertical jumping motions were examined in this computer simulation study. Followings are the primary findings: (1) The hip joint was utilized more vigorously in the horizontal jumping. (2) The joint flexor muscles were activated to a greater level during the countermovement in the horizontal jumping with an effect of moving the body's center of mass in the forward direction. (3) The muscular work was transferred to mechanical energy of the body's center of mass more effectively in the horizontal jump, which resulted in a greater energy gain of the body's center of mass throughout the motion. These differences seem to be caused from the requirement that the body's center of mass needs to be located above the feet in a vertical jumping, whereas this requirement is not so strict in a horizontal jumping.

## Competing interests

The author(s) declare that they have no competing interests.

## Authors' contributions

AN constructed the simulation model, performed the simulation and drafted the manuscript. TK and SF examined the simulated outputs in comparison with the experimental data. TK and SF also did substantial contributions in the process of manuscript preparation. All authors read and approved the final manuscript.
